# Round-window delivery of neurotrophin 3 regenerates cochlear synapses after acoustic overexposure

**DOI:** 10.1038/srep24907

**Published:** 2016-04-25

**Authors:** Jun Suzuki, Gabriel Corfas, M. Charles Liberman

**Affiliations:** 1 Department of Otology and Laryngology, Harvard Medical School, Boston MA 02115, USA.; 2 Eaton-Peabody Laboratories, Massachusetts Eye & Ear Infirmary, Boston MA 02114, USA.; 3 Department of Otorhinolaryngology-Head and Neck Surgery, Tohoku University Graduate School of Medicine, Sendai, Miyagi 980-8574, Japan; 4 Kresge Hearing Research Institute and Department of Otolaryngology—Head and Neck Surgery, University of Michigan, Ann Arbor, MI, USA

## Abstract

In acquired sensorineural hearing loss, such as that produced by noise or aging, there can be massive loss of the synaptic connections between cochlear sensory cells and primary sensory neurons, without loss of the sensory cells themselves. Because the cell bodies and central projections of these cochlear neurons survive for months to years, there is a long therapeutic window in which to re-establish functional connections and improve hearing ability. Here we show in noise-exposed mice that local delivery of neurotrophin-3 (NT-3) to the round window niche, 24 hours after an exposure that causes an immediate loss of up to 50% loss of synapses in the cochlear basal region, can regenerate pre- and post-synaptic elements at the hair cell / cochlear nerve interface. This synaptic regeneration, as documented by confocal microscopy of immunostained cochlear sensory epithelia, was coupled with a corresponding functional recovery, as seen in the suprathreshold amplitude of auditory brainstem response Wave 1. Cochlear delivery of neurotrophins in humans is likely achievable as an office procedure via transtympanic injection, making our results highly significant in a translational context.

Recent work on noise-induced and age-related hearing loss shows that the most vulnerable elements in the inner ear are not the sensory cells, but their synapses with cochlear nerve terminals^1^. A noise exposure causing a large, but ultimately reversible, elevation of cochlear thresholds, can immediately, and permanently, destroy these synapses, thereby silencing up to 50% of the fibers in the cochlear nerve, despite no immediate or delayed loss of hair cells^1^,^3^. Although this cochlear synaptopathy does not elevate thresholds, the loss of neural channels likely causes difficulties understanding speech in noisy or reverberant environments^2^ and may also cause tinnitus^3^,^4^, the phantom sounds commonly brought on by acoustic overexposure. This type of cochlear synaptopathy has been called “hidden hearing loss”^5^, because the auditory deficits can hide behind a normal threshold audiogram.

In the adult ear, cochlear nerve fibers often degenerate after cochlear insult, including noise damage and ototoxic antibiotics^6^. This degeneration occurs with a variable time course, depending on the nature and severity of the insult; however, the unmyelinated terminal dendrites within the organ of Corti disappear first (within hours to days), followed more slowly by the peripheral axons in the osseous spiral lamina (within days to weeks), and, only on a much slower time course, the cell bodies in the spiral ganglion and their central axons that compose the cochlear nerve (over weeks to months and longer)^7^,^8^,^9^. Given that cochlear implants can continue to provide useful hearing for years after hair cell loss, these long-surviving neurons must remain electrically excitable and appropriately connected to their central targets^10^. Thus, in many types of sensorineural hearing loss, there is a long therapeutic window wherein a treatment to elicit neurite outgrowth could reconnect silenced cochlear ganglion cells with hair cells, and thereby potentially improve speech in noise performance and reduce tinnitus.

Neurotrophin-3 (NT-3) is a member of the neurotrophic factor family that contributes to neuronal differentiation, survival and axonal outgrowth via its interactions with TrkC receptors^11^,^12^. Neurotrophins are necessary for normal development of cochlear innervation^13^,^14^,^15^,^16^, and NT-3 is necessary for the formation and maintenance of hair cell ribbon synapses in the postnatal cochlea^17^. In the early postnatal ear, NT-3 is broadly expressed in the organ of Corti, but becomes restricted to the inner hair cells (IHCs) with a longitudinal gradient (apex > base) in the adult^18^,^19^.

Prior studies have shown that exogenous neurotrophins, delivered directly to the cochlear fluids, can prolong the survival of cochlear neurons after hair cell destruction by ototoxic drugs^20^. Furthermore, using transgenic overexpression of NT-3 by supporting cells in mice, we have shown partial synapse regeneration and partial recovery of cochlear neural responses after noise damage^17^. However, a more clinically relevant question is whether exogenous NT-3 can be locally delivered to the inner ear to reverse cochlear synapthopathy. Here, we show that NT-3, delivered at the round window, can regenerate hair cell synapses and restore sound-evoked neural function after a synaptopathic noise exposure. We also describe techniques for surgically accessing, and delivering drugs with a thermoreversible hydrogel to the round window in mice, without jeopardizing cochlear function. Considering that round-window drug delivery in humans could be accomplished via injection through the eardrum^21^, our results suggest a promising therapeutic approach for the treatment of cochlear synaptopathy, and for the tinnitus and auditory processing deficits it likely causes.

## Results

### Temporary vs. permanent threshold shifts and hair cell loss

Prior work on noise-induced cochlear damage in mice has shown that exposure to a mid-frequency noise band for 2 hrs at intensities near 100 dB sound pressure level (SPL) can cause a permanent loss of cochlear-nerve synapses throughout the basal half of the cochlea, without causing significant permanent threshold elevations or loss of hair cells^1^,^22^. We used this type of neuropathic noise exposure in the present study to assess the efficacy of round-window NT-3 delivery in reversing cochlear synaptopathy.

**Figure 1:**
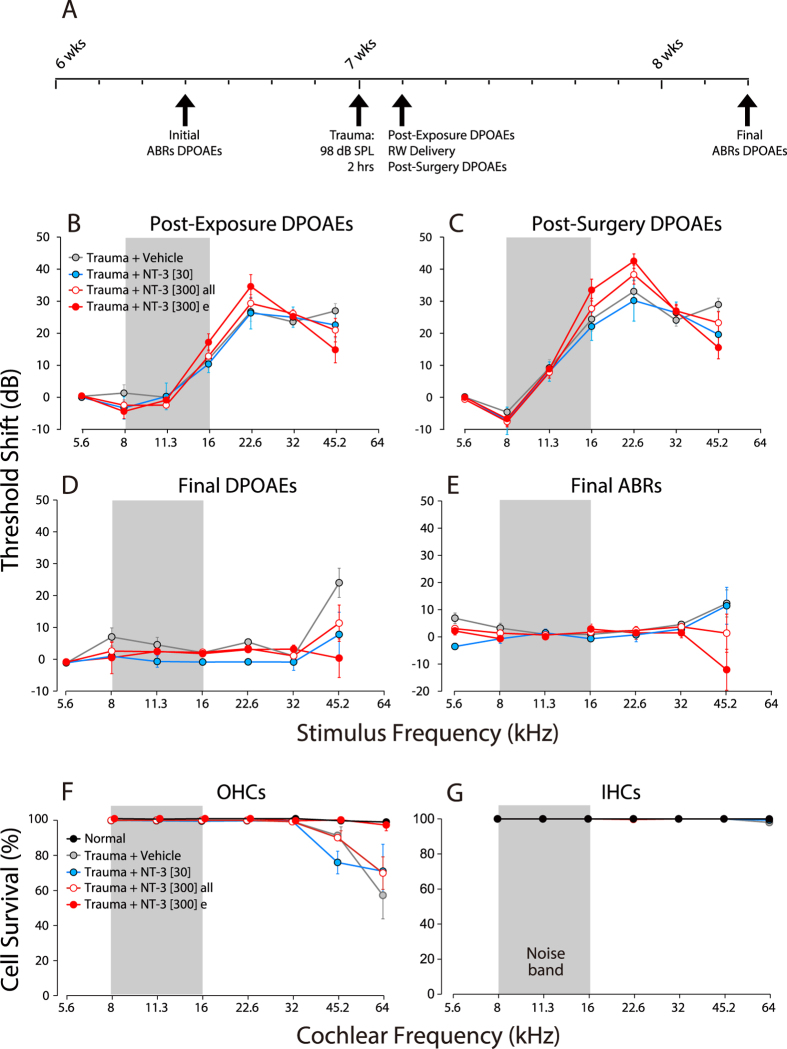
Exposure to octave-band noise at 98 dB SPL for 2 hrs causes a transient threshold elevation, which recovers in most frequency regions and causes minimal hair cell loss. **(A)** Schematic timeline of the experimental protocol, with animal age indicated. (**B,C**) Mean DPOAE threshold shifts (±SEMs) for each group, as measured immediately before and after, respectively, the surgery to expose the round window. (**D,E**) Mean DPOAE and ABR threshold shifts, respectively, as measured 9 days post exposure. Threshold shift is defined relative to the mean thresholds for the same animals measured 2–4 days pre-exposure. (**F,G)** Mean hair cell loss (±SEMs) for each group. Group sizes were as follows: Normal, n = 7; Trauma + Vehicle, n = 11; Trauma + NT-3[30] , n = 7. In (**B**,**C)** the group size for Trauma + NT-3[300] was n = 15. In panels **B–G**, a subgroup of the high-dose NT-3 group, “Trauma + NT-3[300] e” has been separated out, as defined in Fig. 3B. Symbol keys in each row apply to both panels in the same row.

We measured cochlear function before and after the noise exposure and round-window drug delivery, using both auditory brainstem responses (ABRs) and distortion product otoacoustic emissions (DPOAEs) (Fig. 1A). As shown in Fig. 1B, the noise exposure causes ~30 dB of temporary threshold shift at frequencies above the noise band, when measured 24 hrs after the exposure, as expected from prior studies^1^,^22^. Immediately after the DPOAE measures at 24 hrs post-exposure, we opened the otic bulla to reveal the round window for delivery of the slow-release gel, with or without NT-3. The surgical procedure, including the placement of gel on the round window, caused only a modest further increase in thresholds (Fig. 1C), with no significant differences between experimental groups (P = 0.563 by two-way ANOVA).

Nine days later, we re-measured cochlear function by both ABRs and DPOAEs (Fig. 1D,E): thresholds had returned to normal by both measures at all frequencies except the highest tested (45.2 kHz). By both tests, ears receiving the NT-3, whether high (300 ng/μl) or low (30 ng/μl) dose, showed slightly more complete recovery; however, the differences were not statistically significant (P = 0.066 in Fig. 1D and 0.138 in Fig. 1E by two-way ANOVA). Immediately after the final cochlear function test, the cochleae were harvested and fixed for confocal analysis. Hair cell counts showed no loss of inner hair cells (Fig. 1G) and moderate (up to 40%) loss of outer hair cells, but only in the basalmost regions of the cochlear spiral of some groups (Fig. 1F). Interestingly, a subset of the high-dose NT-3 group (filled red symbols) showed minimal outer hair cell (OHC) loss (Fig. 1F) and minimal permanent threshold shift (Fig. 1D,E). This subset was defined on the basis of synaptic regeneration, as described in the next section.

### Synaptic loss and regeneration in the inner hair cell area

**Figure 2:**
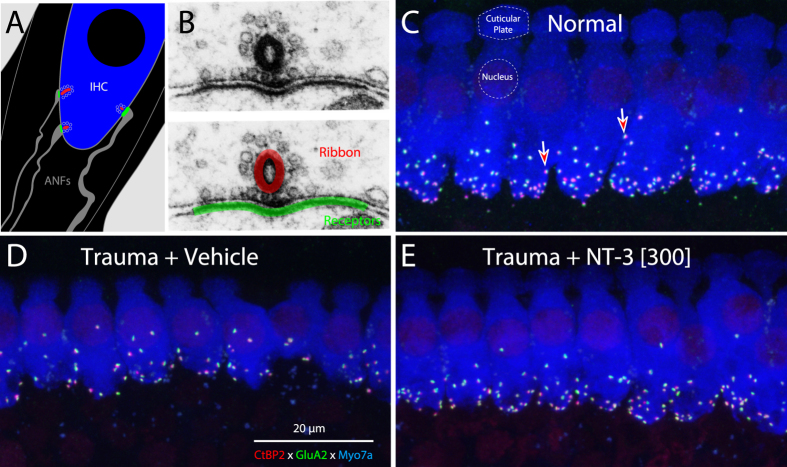
Immunostaining for pre- and post-synaptic markers reveals the noise-induced loss of afferent synapses in the inner hair cell area. (**A**) Schematic of the basolateral region of an inner hair cell (IHC), and three of the 10–20 auditory nerve fibers (ANFs) normally forming ribbon synapses there. (**B**) Electron micrographs showing a ribbon synapse (upper) and a schematic (lower) overlaying the red and green immunostaining for CtBP2 (ribbons) and GluA2 (AMPA-type glutamate receptors) used in the confocal analysis. (**C–E**) Maximal projections of the IHC area immunostained for CtBP2 (red), GluA2 (green) and myosin 7a (blue) to reveal the synapses between auditory nerve terminals and the IHCs. All z-stacks are from the 32 kHz region. The positions of two synapses are noted in **C** (arrows), and the location of one cuticular plate and one IHC nucleus are shown by dashed lines. Scale bar in (**D)** applies to all three panels. Panels A,B are modified from previous publications, e.g. 60.

Each bipolar spiral ganglion neuron (SGN) sends a long central axon to the cochlear nucleus and a shorter peripheral axon to the organ of Corti, where it synapses with hair cells. The vast majority of these ganglion cells (95%) are myelinated, and their peripheral processes are unbranched^23^,^24^. As schematized in Fig. 2A,B, each of these “type-I” SGNs contacts a single inner hair cell by a single terminal swelling, which forms a single ribbon synapse^25^. Thus, we can estimate the number of functional SGNs by counting the pairs of pre-synaptic ribbons and post-synaptic glutamate-receptor patches in the inner hair cell area. In maximum projections from confocal z-stacks, these synapses appear as closely juxtaposed pairs of pre-synaptic CtBP2-positive (red) and post-synaptic GluA2-positive (green) puncta studding the basolateral surface of the inner hair cell (Fig. 2C–E).

**Figure 3:**
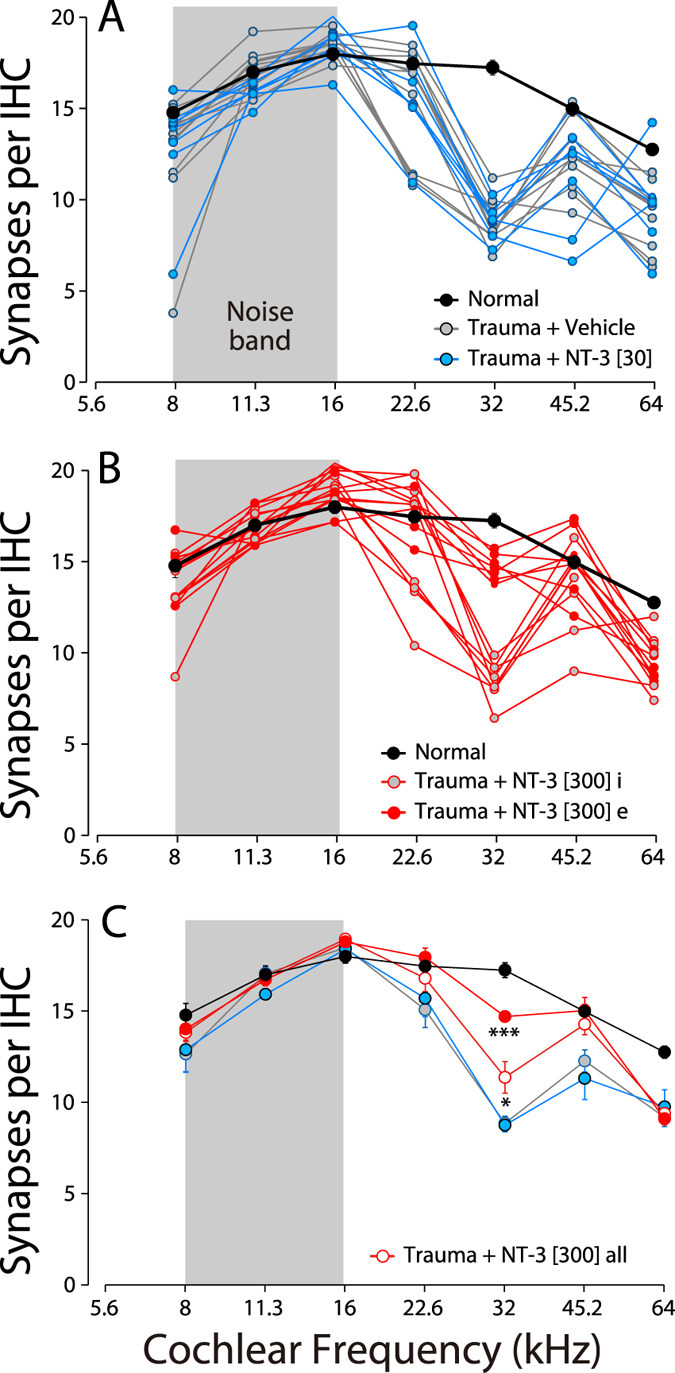
NT-3 delivery can rescue the noise-induced loss of IHC synapses. **(A,B)** Mean normal synaptic counts (±SEMs) are compared to counts in individual animals from different experimental groups, as indicated in the symbol keys. The NT-3 [300] group (**B**) is arbitrarily divided into an effective (**e**) group (n = 7) and an ineffective (**i**) group (n = 8) based on the bimodal distribution of synaptic counts at 32 kHz, the region of maximum synaptopathy in exposed ears treated with vehicle only (**A**). **(C)** Mean synaptic counts (±SEMs) for all the groups shown in (**A**,**B)**. The statistical significance of some of the key intergroup comparisons are shown by asterisks: *P < 0.05; ***P < 0.001.

The loss of inner hair cell synapses is clearly visible in immunostained cochlear tissue from noise-exposed ear (e.g. Fig. 2D), and the synaptic rescue can be seen in the noise-exposed ear treated with high-dose NT-3 (Fig. 2E). The synaptic loss and recovery is shown quantitatively in Fig. 3, where the mean synaptic counts in normal ears are compared to data from individual animals (Fig. 3A,B) and to means from the noise-exposed groups (Fig. 3C). In the vehicle-only ears (Fig. 3A,C), the pattern and extent of synapse loss is similar to that described in prior studies of neuropathic noise: little change in the apical half (≤16 kHz), significant loss in the basal half of the cochlea (≥22.6 kHz) and maximal loss at 32 kHz. The mean loss in the low-dose NT-3 group was statistically indistinguishable from that in the vehicle-only group (P = 1.0 at 32 kHz by one-way ANOVA). In the high-dose NT-3 ears, synaptic counts were significantly higher than those in the vehicle-only groups (P = 0.045 at 32 kHz by one-way ANOVA). Examination of the individual high-dose cases suggests a bimodal distribution of synaptic counts at 32 kHz (Fig. 3B): in roughly half the cases, there was near complete synaptic rescue, whereas, in the other half, there was little effect of the treatment. The mean synaptic counts in the “effective” (e) high-dose subgroup were not significantly different than the normal group (P = 0.188 at 32 kHz by one-way ANOVA), but were dramatically higher than in the vehicle only group (P < 0.0001 at 32 kHz by one-way ANOVA). At 45.2 kHz, the mean synaptic counts in the “effective” (e) high-dose subgroup were also higher than in the vehicle-only group (P = 0.0913 by one-way ANOVA), and were significantly higher than the low-dose NT-3 group (P = 0.0208 by one-way ANOVA). The 24-hr post-exposure threshold shifts at 32 kHz in the effective subset were not different from those in the ineffective subset of the high-dose NT-3 group (Fig. 1B).

**Figure 4:**
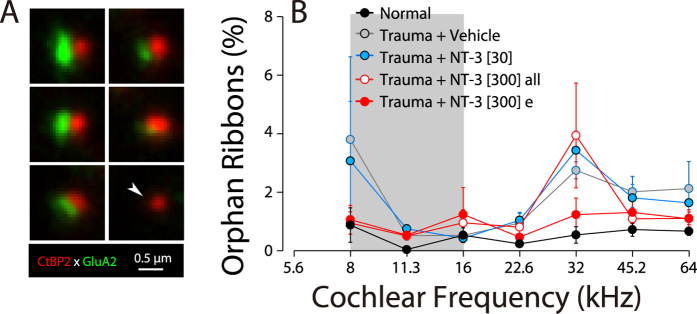
NT-3 delivery can reverse the noise-induced increase in the percentage of orphan synaptic ribbons in the maximum damage region (32 kHz). (**A**) Thumbnail images of six synaptic complexes, showing five with paired ribbons and glutamate receptor patches, and one (white arrowhead) with an orphan ribbon. (**B**) Mean percentages (±SEMs) of orphan ribbons, i.e. unpaired with a closely juxtaposed glutamate-receptor patch for each of the five groups. Group sizes are as given in Figs 1 and 3.

In normal ears, there is an almost perfect pairing of pre-synaptic ribbons and post-synaptic receptor patches: fewer than 1% of ribbons are “orphan”, i.e. unpaired with a receptor patch (Fig. 4). Immediately after neuropathic noise, there are fewer ribbons than normal, and many of the remaining ribbons are unpaired “orphans” However, by 1 wk post exposure, most remaining ribbons are again paired with receptor patches, though the total number of synapses is still far less than normal^17^,^22^. We observed fewer orphan ribbons in the effective high-dose NT-3 subgroup than in either of the other noise-exposed groups in the present study (Fig. 4B), however, the differences did not reach statistical significance. The important point in the present context is that virtually all of the regenerated pre-synaptic ribbons in the effective high-dose NT-3 subgroup are coupled with post-synaptic glutamate receptor patches.

### Synaptic regeneration and functional recovery of suprathreshold responses

Noise induced cochlear synaptopathy does not raise DPOAE thresholds, because DPOAEs are mechanical distortions produced and amplified by the normal function of OHCs, and, as such, don’t require synaptic transmission from IHCs to SGNs for their generation. Although ABRs obviously require SGNs for their generation, noise-induced neuropathy does not raise ABR thresholds, because it is the subgroup of SGNs with high thresholds and low spontaneous discharge rates (SRs) that are preferentially destroyed by noise^26^.

**Figure 5:**
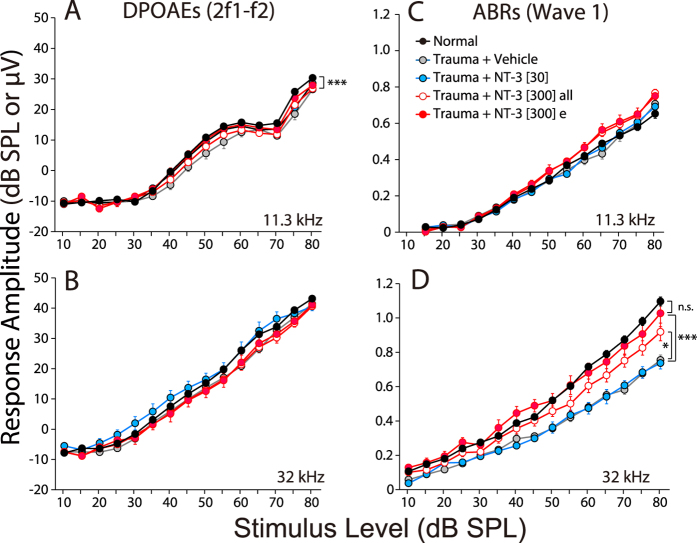
NT-3 delivery can rescue the suprathreshold amplitudes for ABR Wave 1 in the maximum damage region (32 kHz). (**A,B**) Mean amplitude-vs.-level functions (±SEMs) for DPOAEs evoked by primary tones (f1) at 11.3 or 32 kHz, respectively. (**C,D**) Mean amplitude-vs.-level functions (±SEMs) for ABR Wave 1, elicited by tone pips at 11.3 or 32 kHz, respectively. Key in **C** applies to all panels. Group sizes are as given in Figs 1 and 3. The statistical significance of some of the key intergroup comparisons (*re* the vehicle-only group) are shown by asterisks: *P < 0.05; ***P < 0.001.

**Figure 6:**
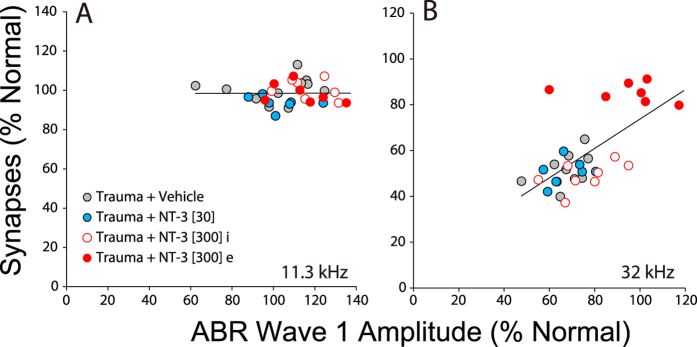
Ears with the most synaptic rescue tended to have the largest ABR Wave 1 amplitudes. (**A,B**) ABR Wave 1 amplitudes are averaged in each ear for levels from 60–80 dB SPL (inclusive) at either 11.3 kHz (**A**) or 32 kHz (**B**), then normalized with respect to the mean values in all animals pre-exposure. Synaptic counts are normalized with respect to mean values in age-matched, unexposed controls. Correlation coefficients for the two panels were 0.0064 (P = 0.9724) (**A**) and 0.6541 (P < 0.0001) (**B**).

The functional measure that can reveal cochlear synaptopathy is the suprathreshold amplitude of wave 1 of the ABR^1^, which represents the summed activity of the SGNs. As shown in Fig. 5, the suprathreshold amplitudes of the ABRs (and DPOAEs) fully recover in all noise-exposed groups at 11.3 kHz, where there is no permanent loss of cochlear synapses (Fig. 3). In contrast, at 32 kHz, where the maximal noise-induced synaptopathy is seen (Fig. 3), the suprathreshold amplitudes are reduced in the vehicle-only and the low-dose NT-3 group by ~40%, which closely matches the 50% drop in synaptic counts (Fig. 3). Matching the regeneration of synapses in the high-dose NT-3 group, especially the “effective” subset, the ABR suprathreshold amplitudes at 32 kHz also showed almost complete recovery, indeed the effective subset of the high-dose group is statistically indistinguishable from normal (P = 0.844 by two-way ANOVA for all levels from 60–80 dB). When viewed on an ear-by-ear basis, there was a good correlation between the set of high-dose NT-3 ears that showed synaptic recovery and those that showed ABR recovery (Fig. 6), reinforcing the notion that IHC synapse counts are the functionally important structural change that underlie the electrophysiologic endpoints.

## Discussion

### Neurotrophins in the Inner Ear

The neurotrophins NT-3 and brain-derived neurotrophic factor (BDNF) are necessary for normal development of the innervation of the inner ear^14^,^27^. In mice with constitutive deletion of both NT-3 and BDNF, or their receptors TrkB and TrkC, hair cells develop normally, but their neural connections are dramatically attenuated, both the sensory fibers in the VIIIth nerve and the efferent feedback pathways from the superior olivary complex (for review see^19^). During inner ear development, NT-3 and BDNF are expressed by supporting cells and hair cells in a decreasing gradient from apex to base^13^,^18^. In the postnatal inner ear, both neurotrophins continue to be expressed^28^,^29^; however, recent studies of transgenic mice with forced over- and underexpression of NT-3 or BDNF in hair cells or supporting cells suggest that NT-3 expression is more critical for the formation and maintenance of cochlear innervation, while BDNF is more critical for the innervation of the vestibular epithelia^17^,^30^.

Prior studies have shown that cochlear perfusion, or round-window delivery, of BDNF and/or NT-3 can prolong the survival of spiral ganglion cells after drug-induced cochlear damage with aminoglycoside antibiotics^31^,^32^,^33^. Furthermore, several studies have documented post-treatment extension of peripheral axons within the osseous spiral lamina and along the denuded basilar membrane with cochlear perfusion of neurotrophins^34^ or neurotrophin gene delivery via adeno-associated virus vectors^35^. In these ototoxicity models, the drug dosage was adjusted to produce total hair cell destruction, thus the peripheral processes have no appropriate targets to reconnect with, and the spiral ganglion cell survival is functionally important only with implantation of a cochlear prosthesis.

### Regeneration vs. Repair of Hair Cells and Synapses

For many years, the dogma in acquired sensorineural hearing loss was that cochlear nerve degeneration only occurred secondarily to hair cell death. Now, we know that if hair cells are destroyed without an external insult, such as noise or ototoxic drugs, cochlear nerve terminals can survive after hair cell loss, so long as supporting cells survive^36^. Furthermore, in noise-induced^1^, age-related^37^ and ototoxic drug damage^38^, cochlear nerve synapses degenerate in large numbers even when hair cells survive. This paradigm shift, coupled with recent data suggesting that NT3 regulates synaptogenesis on postnatal inner hair cells^17^, inspired us to ask whether neurotrophin treatments could reverse cochlear synaptopathy and restore cochlear function in cases where the hair cell targets remain intact. The primary degeneration of cochlear nerve synapses will eventually lead to death of the spiral ganglion cells in the absence of intervention^1^, however, the death of the cell body and central axon takes months to years^1^, during which time the neurons likely remain electrically excitable, given that cochlear implants continue to function for years after hair cell loss. Thus, there may be a long therapeutic window within which to regenerate the peripheral axons and/or synaptic connections in cases where the hair cells remain intact.

In noise-induced cochlear synaptopathy of the type produced here, confocal analysis shows that loss of pre- and post-synaptic puncta is nearly complete by the end of the exposure^22^: synaptic counts at 0 hrs are similar to those seen at 24 hrs, 1 wk and 8 wks post exposure^1^. At even longer post-exposure survivals, the synaptic loss only increases, as age-related changes add to those induced by the noise^39^. When noise-exposed ears are examined by electron microscopy, immediately after exposure, the inner hair cell area shows swollen cochlear nerve terminals, lacking cytoplasm and with broken membranes^40^,^41^. The fact that similar neural ultrastructural damage is after cochlear perfusion of glutamate agonists, and that the effect can be blocked with glutamate antagonists, has suggested that noise-induced neuropathy is a type of glutamate excitotoxicity at this highly active synapse^42^. When cochlear explants are exposed to glutamate agonists *in vitro*, inner hair cell ribbons quickly disappear, and the peripheral terminals of the cochlear nerve retract to the first node of Ranvier, in the osseous spiral lamina^43^. Thus, the observation of near-normal synaptic counts in some of the high-dose NT-3 ears nine days post-exposure strongly suggests that the treatment has regenerated peripheral terminals and elicited synaptic connections *de novo*, rather than simply enhancing recovery of damaged neuronal terminals.

In our hands, delivery of the NT-3 treatment yielded bimodal results (Fig. 3B), with roughly a 50% “success rate”. We attribute the failures to the difficulty in delivering a viscous gel through a small micropipette (tip diameter ~ 150 microns), as constrained by the small size of the mouse round window and the small size of the bulla opening required to minimize surgically induced threshold shifts. These constraints forced us to increase poloxamer dilutions to levels that compromised its gelling properties and likely decreased the contact time of the injectate with the round window membrane as it became diluted with exudate from the surrounding tissue and diffused away from the injection site. This, in turn, likely decreased the effective dose of NT-3 delivered to the cochlear fluids.

The special vulnerability of hair cells in the basal tip, or “hook” of the cochlear spiral (Fig. 1) has been observed after acoustic overexposure in chinchilla, cat, guinea pig and mouse^9^,^44^,^45^,^46^. The phenomenon is not well understood: for example, it cannot be explained based on measurements of cochlear mechanical motion^47^. The NT-3 mediated rescue of the noise-induced OHC lesions in the hook (Fig. 1F) is consistent with prior reports^48^,^49^. Studies of the dynamics of hair cell death after noise suggest that, for exposures similar in severity to those used here, there is significant ongoing hair cell death between 24 hrs and 14 days post-exposure^46^. Considering that NT-3 has been reported to have protective effects on hair cell survival^48^,^49^ (and that there is no evidence suggesting a role for NT-3 in hair cell regeneration), the beneficial effects of NT-3 delivery at 24 hrs post exposure likely represent enhanced recovery of damaged hair cells rather than regeneration of missing hair cells. Given that, adult hair cells can survive indefinitely after complete cochlear nerve degeneration subsequent to central axotomy^50^, the hair cell rescue is likely a direct effect of the NT-3, rather than an indirect effect through enhanced synaptic connection. However, the mechanism is unclear, since in the adult cochlea only spiral ganglion neurons and their processes express Trk receptors^19^.

### Application to Human Hearing Impairment

Both hair cells and cochlear nerve fibers are required for normal hearing. Damage to hair cells elevates the sound pressure required for stimulus detection. This “threshold shift” is well measured by conventional pure-tone audiometry, and is what has conventionally been thought of “hearing loss”. Damage to cochlear nerve fibers, *per se*, will not elevate audiometric thresholds unless the loss is catastrophic, i.e. >90%^51^,^52^); however, the loss of functional neural channels will reduce the intelligibility of complex sounds like speech, even when they are detectable. Complementing the animal work showing that cochlear synapses are the most vulnerable elements in noise-damage^1^, aging^37^ and aminoglycoside otototoxicity^38^, a recent study of aging humans suggests that loss of synaptic connections between spiral ganglion cells and surviving hair cells is an important aspect of presbycusis^53^, and that this cochlear neuropathy may be a major cause of the most common hearing-related complaint of the elderly, i.e. difficulties understanding speech in a noisy environment^54^.

The idea that a significant, but ultimately reversible, noise-induced threshold shift can be associated with a 50% loss of cochlear nerve synapses has also provided a new hypothesis for the origins of tinnitus, the phantom perception of sound that is often the permanent result of a noise exposure even if the threshold elevation is transient^55^. It has been argued that the permanent loss of spontaneous and sound-driven activity in a subgroup of cochlear nerve fibers leads to a central gain readjustment that drives hyperactivity in central auditory pathways and thereby causes tinnitus^5^.

Thus, there is likely a significant clinical population with some degree of hearing impairment (or tinnitus) that could be addressed by a treatment analogous that used here in noise-exposed mice^56^. The present experiments show that round window delivery can be effective local access route to the inner ear, even for a protein as large as NT-3 (13.6 kD molecular weight). Here, we used a poloxamer, a thermoreversible hydrogel that is liquid at 4 °C but gels at body temperature, to extend the drug delivery time^57^, which is important for a molecule like NT-3 with a short half-life once it enters body fluids^58^. Prior studies have seen biological effects of neurotrophins delivered to the round window using a variety of slow-release strategies^59^.

Although the success of local NT-3 delivery in regenerating cochlear synapses when administered 24 hrs post exposure is an important proof of concept, many key questions remain, most importantly how long after the insult can neurite extension and synaptogenesis still be elicited.

## Methods

### Animals and experimental design

CBA/CaJ male mice were obtained from Jackson Laboratories at 6 wks of age. After brief acclimatization to our vivarium, cochlear function was tested by measurement of ABRs and DPOAEs. Animals were then randomly assigned to one of four experimental groups. One group (n = 7) survived for two weeks without noise exposure or surgery to assess normal cochlear synaptic counts. Three other groups were exposed to a noise band designed to destroy cochlear synapses^22^; 24 hrs later cochlear function was tested again via DPOAEs, and the left bulla was opened to access the round window membrane, onto which a solution was placed containing either: 1) 300 ng/μl NT-3 in poloxamer (n = 15), 2) 30 ng/μl NT-3 in poloxamer (n = 7), or 3) poloxamer only (n = 11). Immediately after closing the wound, cochlear function was re-tested to assess the effects of the surgical manipulations. The animals were returned to the vivarium for 8 days, after which final cochlear function tests were performed at 9 days after noise exposure, and the cochleae were fixed by intracardiac perfusion and removed for histological analyses. All experimental procedures were approved by the Institutional Animal Care and Use Committee of the Massachusetts Eye and Ear Infirmary and conducted in accordance with the NIH Guide for the Care and Use of Laboratory Animals.

### Noise exposure

Mice were exposed, awake and unrestrained, to octave-band noise (8–16 kHz) for 2 hrs at 98 dB sound pressure level (SPL) in a reverberant sound-exposure box. Mice were placed within acoustically transparent wire cages on a rotating platform. The noise waveform was generated digitally using a fifth-order Butterworth filter, amplified through a power amplifier (Crown D75A), and delivered by a loudspeaker (JBL2446H) coupled to an exponential horn in the roof of the box. Sound levels were verified in the center of the cage with a ¼′′ Bruel and Kjaer condenser microphone before each exposure and varied by less than 1 dB in the cage space.

### Cochlear function testing

ABRs and DPOAEs were recorded as described previously^1^. The animals were anesthetized with an intraperitoneal injection of ketamine (100 mg/kg) and xylazine (20 mg/kg) and placed in an acoustically and electrically shielded room maintained at 32 °C. Acoustic stimuli were delivered through a custom acoustic system consisting of two miniature dynamic earphones used as sound sources (CDMG15008-03A, CUI) and an electret condenser microphone (FG-23329-PO7, Knowles) coupled to a probe tube to measure sound pressure near the eardrum (for details see http://www.masseyeandear.org/research/ent/eaton-peabody/epl-engineering-resources/epl-acoustic-system/).

Custom LabVIEW software controlling National Instruments 24-bit soundcards (6052E) generated all ABR/DPOAE stimuli and recorded all responses. For ABRs, stimuli were 5 ms tone pips (0.5 ms cos^2^ rise-fall) at frequencies from 5.66 to 45.25 kHz (in half-octave steps) delivered in alternating polarity at 35/s. Electrical responses were collected via needle electrodes at the vertex and at the ventral edge of the pinna with a ground reference near the tail, amplified 10,000X with a 0.3–3 kHz passband, and averaged with 1024 responses at each SPL. Responses were collected for stimulus levels in 5-dB steps from 10 dB below threshold up to 80 dB SPL. ABR threshold was defined as the lowest sound level at which a reproducible waveform could be observed. When there was no detectable response at 80 dB SPL, threshold was defined as 85 dB. Wave 1 amplitude was defined as the difference between the average of the 1-ms pre-stimulus baseline and the wave 1 peak (P1), after additional filtering to remove low-frequency baseline shifts. For DPOAEs, the cubic distortion product 2*f*_1 _− *f*_2_ was measured in response to primaries f_1_ and f_2_ (frequency ratio *f*_2_/*f*_1_ = 1.2, and level ratio L1 = L2 + 10), where f_2_ varied from 5.66 to 45.25 kHz in half-octave steps. Primaries were swept in 5 dB steps from 10 to 80 dB SPL (for *f*_2_). The DPOAE at 2*f*_1 _− *f*_2_ was extracted from the ear canal sound pressure after both waveform and spectral averaging. DPOAE threshold was computed by interpolation as the primary level (*f*_1_) required to produce a DPOAE of 0 dB SPL.

### NT-3 Formulation

Lyophilized recombinant human NT-3 (100 μg) (#450-03, PeproTech) was reconstituted in 100 μL of 10 mM phosphate-buffered saline (PBS) at pH 7.3 with 0.1% bovine serum albumin (BSA), yielding 100 μL of a stock solution with a NT-3 concentration of 1 mg/ml, frozen and stored at −80 °C until used. A 24% (w/w) stock solution of poloxamer 407 (#16758, Sigma-Aldrich) was prepared by slowly adding it to cold 10 mM PBS, with Evans blue dye (50 ppm), and sterilized by filtration. Immediately before the drug administration surgery, 5 μL of NT-3 stock solution was mixed with 11.7 μL of the 24% poloxamer stock solution or 117 μL of the 24% poloxamer stock solution and 45 mL of 10 mM PBS to obtain 17% poloxamer containing either 300 ng/μL or 30 ng/μL NT-3. This 17% poloxamer 407 solution is liquid in the refrigerator or at room temperature, but becomes highly viscous at body temperature.

### Round-window administration of NT-3

Mice were anesthetized with an intraperitoneal injection of ketamine (100 mg/kg) and xylazine (20 mg/kg) and positioned right ear down. A 12-mm postauricular skin incision was made, and subcutaneous tissues and superficial fascia of the neck were bluntly dissected. After exposing the otic bulla, tympanotomy was performed using a 0.6 mm diamond burr, and the hole was enlarged using microforceps until the round window membrane was clearly visible. A pulled glass micropipette (P0674-1PAK, Sigma-Aldrich) was positioned within the round window niche, and 1 μl of the poloxamer solution was injected using UltraMicroPump 3 (World Precision Instruments). The tympanotomy hole was sealed with muscle, and the wound was closed with 4-0 vicryl sutures (Ethicon).

### Cochlear processing and immunohistochemistry

After transcardial perfusion with 4% paraformaldehyde, cochleae were perfused through the cochlear scalae, dissected from the temporal bones, and post-fixed for 2 hrs at room temperature. Cochleae were then decalcified in 0.12 M EDTA at room temperature for 2 days and microdissected into 6 pieces for whole-mount cochlear processing. For immunostaining, cochlear pieces were blocked with 5% normal horse serum in PBS and 0.3% Triton X-100 for 1 hr at room temperature followed by overnight incubation at 37 °C with following primary antibodies diluted in 1% normal horse serum with 0.3% TX: 1) mouse (IgG1) anti-CtBP2 (C-terminal Binding Protein) at 1:200 (#612044, BD Transduction Labs) for quantifying pre-synaptic ribbons; 2) mouse (IgG2a) anti-GluA2 (Glutamate receptor subunit A2) at 1:2000 (#MAB397, Millipore) for quantifying post-synaptic receptor patches; and 3) rabbit anti-Myosin 7a at 1:200 (#25-6790 Proteus Biosciences) for delineating hair cells. Cochlear pieces were washed and then incubated twice for 60-min at 37 °C in species-appropriate secondary antibodies: 1) Alexa Fluor 488-conjugated goat anti-mouse (IgG2a) at 1:1000 (#A21131, Life Technologies); 2) Alexa Fluor 568-conjugated goat anti-mouse (IgG1) at 1:1000 (#A21124, Life Technologies); 3) Alexa Fluor 647-conjugated chicken anti-rabbit at 1:200 (#A21443, Life Technologies). Finally, cochlear pieces were slide mounted using Vectashield (Vector Labs) and coverslipped. Cochlear pieces were imaged at low magnification (x4 objective) using a fluorescent microscope (E800, Nikon), and a custom ImageJ plug-in http://www.masseyeandear.org/research/otolaryngology/investigators/laboratories/eaton-peabody-laboratories/epl-histology-resources/) was used to create a cochlear frequency map.

### Synapse and hair cell counts

Immunostained cochlear whole mounts were imaged at 7 log-spaced cochlear frequency regions via confocal microscopy (TCS SP5, Leica) with a glycerol-immersion 63X objective (1.3 N.A.) and 3.2X digital zoom. For each image stack, the z dimension was sampled at 0.25 μm, with the span adjusted to include all synaptic elements in the xy field of view. Each z-stack included 8~12 adjacent inner hair cells, and two contiguous z-stacks were obtained in each frequency location in each ear. To identify and count hair cell synapses, image stacks were ported to image processing software (Amira, Visage Imaging). Pre-synaptic ribbons in each z-stack were isolated and counted using the “*connected components”* function in Amira. Inner hair cells in each stack were counted using the faint nuclear staining from the anti-CtBP2 and hair cell staining from the anti-Myosin 7a. To quantitatively assess the juxtaposition of pre-synaptic ribbons and post-synaptic receptor patches, we used custom software that extracts the voxel space within 1 μm around each ribbon and produces a thumbnail array of these miniature projections, that can be scanned to count synapses versus orphan ribbons. For IHC and OHC counts, we acquired z-stacks at the same 7 log-spaced cochlear frequency locations, with the same glycerol-immersion 63X objective, but without digital zoom, such that each stack contained 20~30 inner hair cells and 60~90 outer hair cells across the three rows. IHC and OHC survival was assessed using the anti-Myosin7 immunostain.

### Statistical analysis

Statistical analyses were conducted using Kaleidagraph (Synergy Software) for one-way analysis of variance (ANOVA), followed by a post hoc Tukey HSD test or Matlab (MathWorks) for two-way ANOVA, followed by a post hoc Tukey HSD test. All of the data are presented as means ± standard errors of the mean (SEMs). Correlation coefficients between ABR P1 amplitudes and synapse numbers per IHC were calculated using the Pearson product-moment correlation coefficient.

## Additional Information

**How to cite this article**: Suzuki, J. *et al*. Round-window delivery of neurotrophin 3 regenerates cochlear synapses after acoustic overexposure. *Sci. Rep.* **6**, 24907; doi: 10.1038/srep24907 (2016).
